# Rhizobia induce SYMRK endocytosis in *Phaseolus vulgaris* root hair cells

**DOI:** 10.1007/s00425-023-04116-0

**Published:** 2023-03-16

**Authors:** Raúl Dávila-Delgado, Karen Flores-Canúl, Marco Adán Juárez-Verdayes, Rosana Sánchez-López

**Affiliations:** Departamento de Biología Molecular de Plantas, Instituto de Biotecnología, Universidad Nacional Autónoma de México, Avenida Universidad 2001, Colonia Chamilpa, 62210 Cuernavaca, Morelos Mexico

**Keywords:** Brefeldin A, *Phaseolus vulgaris*, Root hair, Symbiosis, SYMRK, Tyrphostin A23, YXXØ

## Abstract

**Main conclusion:**

*Pv*SYMRK-EGFP undergoes constitutive and rhizobia-induced endocytosis, which rely on the phosphorylation status of T589, the endocytic YXXØ motif and the kinase activity of the receptor.

**Abstract:**

Legume-rhizobia nodulation is a complex developmental process. It initiates when the rhizobia-produced Nod factors are perceived by specific LysM receptors present in the root hair apical membrane. Consequently, SYMRK (Symbiosis Receptor-like Kinase) becomes active in the root hair and triggers an extensive signaling network essential for the infection process and nodule organogenesis. Despite its relevant functions, the underlying cellular mechanisms involved in SYMRK signaling activity remain poorly characterized. In this study, we demonstrated that *Pv*SYMRK-EGFP undergoes constitutive and rhizobia-induced endocytosis. We found that in uninoculated roots, *Pv*SYMRK-EGFP is mainly associated with the plasma membrane, although intracellular puncta labelled with *Pv*SymRK-EGFP were also observed in root hair and nonhair-epidermal cells. Inoculation with *Rhizobium etli* producing Nod factors induces in the root hair a redistribution of *Pv*SYMRK-EGFP from the plasma membrane to intracellular puncta. In accordance, deletion of the endocytic motif YXXØ (YKTL) and treatment with the endocytosis inhibitors ikarugamycin (IKA) and tyrphostin A23 (TyrA23), as well as brefeldin A (BFA), drastically reduced the density of intracellular *Pv*SYMRK-EGFP puncta. A similar effect was observed in the phosphorylation-deficient (T589A) and kinase-dead (K618E) mutants of *Pv*SYMRK-EGFP, implying these structural features are positive regulators of *Pv*SYMRK-EGFP endocytosis. Our findings lead us to postulate that rhizobia-induced endocytosis of SYMRK modulates the duration and amplitude of the SYMRK-dependent signaling pathway.

**Supplementary Information:**

The online version contains supplementary material available at 10.1007/s00425-023-04116-0.

## Introduction

Over the past 20 years, much effort has been expended in identifying genes involved in plant–microbe interactions (Roy et al. 2020). A feature that soon became evident is the role played by a battery of genes, such as *SYMRK/DMI2/NORK*, *CASTOR, POLLUX, DELLA, CYCLOPS*, among others, as essential components of a common symbiotic signaling pathway (CSSP) involved in plant-mycorrhizal, non-legume-actinobacteria and legume-rhizobia symbiotic interactions (Yang et al. 2022). Although genetics and molecular studies provided insights into the functional role of CSSP genes and the molecular mechanisms regulating their signaling activity, our current understanding of the cellular processes that mediate their functions is still limited (Roy et al. 2020).

*Symbiosis Receptor-like Kinase* gene, also known as *SYMRK* in *Phaseolus vulgaris, Lotus japonicus* and *Arachis hypogaea*, *DMI2* in *Medicago* *truncatula* or *NORK* in *M. sativa*, hereafter named *SYMRK*, is a plant-specific plasma membrane (PM) leucine-rich repeat (LRR) receptor-like kinase gene essential for both fungal and bacterial symbiosis. However, SYMKR functions have been more extensively described in the legume-rhizobia interaction (Endre et al. 2002; Stracke et al. 2002; Markmann et al. 2008; Kosuta et al. 2011; Roy et al. 2020).

Legume nodulation is characterized by the development of a new organ in the root, the nitrogen-fixing nodule, where the bacteria reduce atmospheric nitrogen and provide assimilable nitrogen metabolites for the plant growth, while the legume supplies di-carbon compounds, as an energy source for rhizobia (Roy et al. 2020). The nodule development, a well-coordinated process, is initiated by the mutual sensing of molecular signals secreted by the root (flavonoids) and the rhizobia (lipochito-oligosaccharides named Nod factors), which are specifically recognized by the bacterial transcriptional activator NodD and plant-specific LysM receptor-like kinases (*L. japonicas *NFR1/NFR5 or *M. truncatula* NFP/LYK3), respectively (Roy et al. 2020). It has been established that growing root hairs with typical cytoplasmic streaming in the subapical region are those potentially competent to respond to rhizobia inoculation or Nod factors treatment (Sieberer and Emons 2000). Nod-factor perception triggers molecular and cellular responses, such as the activation of SYMRK, signal transduction cascades, actin cytoskeleton rearrangements, root apical swelling and expression of the common symbiotic signaling pathway (CSSP) genes, among others, all of them required for the setting up of the epidermal infection and cortical cell division (Oldroyd and Downie 2008; Yang et al. 2022). Further, rhizobia get trapped in a groove formed in the curled root hair, creating an infection chamber. At this point, the root hair cell wall and PM invaginate, forming a unique tubular structure, known as the infection thread (IT), through which rhizobia enter the root and invade the cortex. In parallel, the cortical cells re-activate their cell cycle to form a nodule primordium, which progressively differentiates into a mature nodule. Finally, rhizobia are progressively “delivered” from the IT into cells at the central zone of the nodule, within a *quasi*-organelle structure known as a symbiosome, in which the rhizobia differentiate into N_2_-fixing bacteroids (Oldroyd and Downie 2008).*symrk* mutants show a root hair curling-deficient response upon rhizobia inoculation, but root hair swelling, and branching responses were observed. Therefore, epidermal infection and cortical cell division are impaired; accordingly, no nodules are formed (Endre et al. 2002; Stracke et al. 2002; Esseling et al. 2004). Interestingly, the nodulation deficient phenotype of *symrk* mutants is fully restored by the Eurosid versions of SYMRK (*Datisca* *glomerata *and *Tropaeolum majus*), but it is not by the shorter versions of SYMRK from the non-nodulating eudicots *Papaver* *rhoeas*, *Solanum lycopersicum* (formerly *Lycopersicon esculentum*) and the monocots *Oryza sativa* and *Zea mays* (Markmann et al. 2008). A deeper understanding of SYMRK functions in legume-rhizobia symbiosis was achieved using *Sesbania* *rastrata*, *P. vulgaris* and *M. truncatula* transgenic roots expressing a *SYMRK*-specific RNAi. *SYMRK* downregulated roots generate scarce, small, non-infected nodule-like structures (*pseudo-*nodules) that present abundant wide ITs and an inefficient release of bacteria from the IT. Consequently, the nodule cells are poorly infected and symbiosomes are absent, as confirmed by transmission electron microscopy analysis (Capoen et al. 2005; Limpens et al. 2005; Sánchez-López et al. 2011). In addition, *SYMRK*-silencing affects the vascular bundle development, as we have previously described for *P. vulgaris* nodulation (Sánchez-López et al. 2011). The *SYMRK-*silencing phenotype correlates with the spatio-temporal expression pattern of *SYMRK* in the epidermis of uninoculated roots. Additionally, in rhizobia-inoculated roots, the expression of *SYMRK* is detected in the cortical zone in front of the infection site, as well as in the nodule primordium and the central zone of the nodule (Bersoult et al. 2005; Capoen et al. 2005; Limpens et al. 2005; Sánchez-López et al. 2011; Den Herder et al. 2012). Interestingly, *Pv*SYMRK has been immunodetected associated with uninfected cells interspersed in the central tissue of *P. vulgaris* mature nodules, as well as with the nodule vasculature and root central cylinder (Sánchez-López et al. 2011). Together, these data indicate that SYMRK is a key regulator of both infection and nodule organogenesis, opening the question of how SYMRK signaling is transduced in such a diversity of cells expressing the receptor. Some relevant answers were also obtained using biochemical and molecular biology strategies (Yoshida and Parniske 2005; Markmann et al. 2008; Antolín-Llovera et al. 2014a; Saha et al. 2014) which outlined how to approach the cell biology of SYMRK.

Legume *SYMRK* genes encode for polypeptides of 919–926 residues, with a highly conserved sequence that contains a signal peptide sequence, an ectodomain consisting of a malectin-like domain (MLD) linked by a GDPC motif to three LRRs, a single transmembrane domain, and a cytoplasmic kinase domain (Markmann et al. 2008). The function of the ectodomain has not been established, but it seems to be related to SYMRK degradation-mediated signaling and protein–protein interactions, likely with *Lj*NFR5 (Antolín-Llovera et al. 2014a, b; Li et al. 2018). Substitution of the Pro residue in the GDPC motif by a leucine, as in the *Ljsymrk*-14 mutant (Kosuta et al. 2011), appears to abolish a proteolytic release of the malectin-like domain (MLD) fragment and impairs the epidermal infection process, although cortical and nodule primordium infection are apparently unaffected (Kosuta et al. 2011; Antolín-Llovera et al. 2014a; Li et al. 2018). Additionally, the overexpression of the SYMRK cytoplasmic kinase domain induces spontaneous nodulation in the absence of rhizobia (Saha et al. 2014). Biochemical studies on the SYMRK kinase activity and phosphorylation status (“phospho-code”) have been focused on specific motifs, as well as on Ser/Thr residues and the Tyr gatekeeper. In vitro phosphorylation assays, using the *E. coli*-expressed SYMRK intracellular domain, reveal that the individually mutated residues *Lj*SYMRK T593A and *Lj*SYMRK T760A (*A.* *hypogaea* SYMRK T763A), as well as the gatekeeper Tyr (*Ah*SYMRK Y670F/A/E) lead to a significantly reduced autophosphorylation and kinase activities. Moreover, the nodulation *minus* phenotype of *M.* *truncatula* TR25, a *dmi2* null mutant, is partially restored by *Ah*SYMRK (Y670/F/A). Abnormal ITs are formed, but their progression is arrested at the epidermal-cortical interface, although empty nodules are eventually generated (Saha et al. 2016). Hence, the activation of SYMRK is phosphorylation-dependent (Yoshida and Parniske 2005; Samaddar et al. 2013; Saha et al. 2016). Additionally, substituting the catalytic Lys residue in the conserved phosphotransfer VAVK motif (K622 in *Lj*SYMRK) for a Glu residue leads to a loss of kinase activity, meaning *Lj*SYMRK K612E is a kinase-dead (KD) mutant (Yoshida and Parniske 2005; Saha et al. 2014).

Regarding SYMRK subcellular localization, Riely et al. (2013) reported that, in the native root hair cell environment, DMI2-GFP is mainly associated with the PM. In contrast, a significant number of DMI2-GFP-labeled “cytoplasmic organelles” are detected upon Nod factors treatment. However, no additional evidence has been further described. In the infection zone of *M.* *truncatula* nodules, DMI2-GFP seems to be associated with the host cell PM and the membrane surrounding the IT (Limpens et al. 2005).

Of particular interest are three E3 ubiquitin ligase genes (*LjSIE3*, *LjSINA4* and *MtPUB2*) involved in nodulation, which were identified as potential interactors of the intracellular region of SYMRK (Den Herder et al. 2012; Liu et al. 2018). E3 ubiquitin ligase-mediated ubiquitination of PM proteins is a signal that triggers their endocytosis and, eventually, sorting for degradation (Schwihla and Korbei 2020). Additionally, confocal images of *Nicotiana benthamiana* leaf cells co-expressing *Lj*SYMRK and *Lj*SINA4DN have revealed the colocalization and redistribution of both proteins from the PM to dots at the cytosolic interface with the PM (Den Herder et al. 2012). Therefore, it is plausible to consider that SYMRK ubiquitination and further endocytosis are involved in downstream signaling cascade at early stages of nodulation. Several groups have evoked this hypothesis; however, it has not been formally addressed.

Inspired by the current understanding of the relevant role of endocytic steps in the signaling activity of other plant receptor-like kinases (RLKs), such as FLAGELLIN SENSING 2 (FLS2) and BRASSINOSTEROID INSENSITIVE 1 (BRI1), and the auxin transporters PIN (Antolín-Llovera et al. 2014b; Claus et al. 2018), in this study we investigated whether *Pv*SYMRK undergos endocytosis. We analyzed the effect of endocytosis-specific inhibitors and site-directed mutagenesis on the subcellular disttribution of *Pv*SYMRK-EGFP in nonhair-epidermal cells and in rhizobia-responsive root hairs. Our data demonstrate that rhizobia induce the endocytosis of SYMRK in root hairs at early stages of the epidermal infection.

## Materials and methods

### Plants and bacteria growth conditions

*Phaseolus vulgaris* cv. Negro Jamapa (common bean) seeds were obtained from local farmers in Morelos, Mexico. Seeds were surface-sterilized with 70% alcohol (1 min) and 20% commercial chlorine (5 min). The germination was carried out in a sterile tray on an absorbent paper moistened with liquid Fåhraeus medium, at 28 °C for 48 h in the dark. Composite plants with transgenic roots were generated by *A. rhizogenes* K599-mediated transformation, as described by Sánchez-López et al. (2011). Transgenic roots (fluorescent roots) were identified by direct observation of hairy roots under an epifluorescence stereomicroscope (SXZ7, Olympus) looking for the expression of LifeAct-mTurquoise2, as a fluorescent transgenic marker*.* Non-fluorescent roots were eliminated to favor the growth of transgenic roots. Transgenic roots were allowed to recover for two days in liquid Fåhraeus medium. Subsequently, composite plants were used for confocal microscopy analysis or transferred to pots containing vermiculite to further be inoculated with either *Rhizobium tropici* CIAT899 GUS (Vinuesa et al. 2003), *R. etli* CE3 pMP604 (Dávila-Delgado et al. 2020) or *R. etli* CFNX89 (a CE3 derivative strain cured of the symbiotic plasmid, pSym, therefore it does not produce Nod factors; Brom et al. 1992; Corvera et al. 1999) diluted in 10 mM MgSO_4_ to an OD_600_ of 0.05. Plants were grown in a greenhouse with controlled environment conditions (28 °C, 16 h/8 h photoperiod) and watered with nitrogen-free Fåhraeus medium and harvested at the indicated time points. *Agrobacterium rhizogenes* K599 was grown in LB medium supplemented with 200 µg/ml spectinomycin. Rhizobia strains were grown in PY medium supplemented with 7 mM CaCl_2_ and 100 µg ml^−1^ streptomycin, 100 µg/ml spectinomycin (*R. tropici* CIAT899 GUS), 20 µg ml^−1^ nalidixic acid, 100 µg ml^−1^ streptomycin and 5 µg ml^−1^ tetracycline (*R. etli* CE3 pMP604) or 20 µg ml^−1^ nalidixic acid (*R. etli* CFNX89).

### Plasmid constructions

In all cases, PCR amplified fragments were first cloned into the pENTR^™^/D-TOPO^®^ entry vector (Invitrogen, Waltham, Massachusetts, USA) to further be subcloned in the suitable plant expression vector by recombination using Gateway^®^ LR Clonase^™^ II Enzyme Mix (Invitrogen).

All cloning steps were performed in *E. coli* DH5a, confirmed by sequencing, and final constructs were electroporated into *A. rhizogenes* K599. The primer information is listed in Table S1.

### pBGWFS7-pSYMRK

A 1622 bp fragment upstream *SYMRK* start codon was amplified by genomic PCR using the primers pPvSYMRKH5′ UP (which adds a *Hind*III restriction site at the 5′ end of the PCR product) and pPvSYMRK3′ LW and subcloned by recombination in the plant vector pBGWFS7 in order to generate the transcriptional fusion p*PvSYMRK*::*GFP-GUS*.

### pK2GW7-pPvSYMRK::PvSYMRK-EGFP-p35S::LifeAct-mTurquoise2

This plasmid was constructed in three steps: (i) to generate the cassette p35S::*LifeAct-mTurquoise2*, a chimeric cDNA coding for LifeAct-mTurquoise2 (772 pb) was PCR amplified from plasmid mTurquoise2 using the primers LifeAct LUP, which comprises 51 nucleotides coding for the actin-binding domain, known as LifeAct (Vidali et al. 2009), in frame with 15 nucleotides of the 5′ end of the mTurquoise2 coding sequence, and the primer mTurquoise2 LW. *LifeAct-mTurquoise2* cDNA was subcloned by recombination into the vector pK2GW7 to generate the transcriptional fusion p35S::*LifeAct-mTurquoise2* (pK2GW7-*LifeAct-mTurquoise2*). (ii) To construct the cassette p*PvSYMRK*::*PvSYMRK-EGFP* (5152 bp; Fig. S1a), *PvSYMRK* cDNA was PCR amplified from total cDNA *P. vulgaris* roots using the primers 5UTR UP and PvSYMRK LW and cloned by recombination into the plasmid pH7FWG2, creating the cassette p35S::*PvSYMRK-EGFP*-T35S. A *Hind*III restriction site located at 171 pb upstream to *PvSYMRK* start codon (Fig. S1a) was advantageous to substitute the promoter 35S for the *Hind*III fragment from the cassette p*PvSYMRK*::*GFP-GUS* to generate the plasmid pH7FWG2-*pPvSYMRK*::*PvSYMRK-EGFP*. This plasmid was the template for a PCR reaction using the pair of primers MauBIp*Pv*SYMRK UP and T35S_SacI; the PCR product (5,425 bp) was cloned into pENTR^™^/D-TOPO^™^ (Invitrogen), generating pENTR-MauBI_p*PvSYMRK*::*PvSYMRK-EGFP*-T35S_SacI. (iii) To construct the plasmid pK2GW7-p*PvSYMRK*::*PvSYMRK-EGFP*-p35S::*LifeAct-mTurquoise2*, the plasmid pENTR-MauBI_p*PvSYMRK*::*Pv*SYMRK-EGFP-T35S_SacI was digested with *Mau*BI and *Sac*I restriction enzymes and the released fragment was cloned in the corresponding restriction sites in pK2GW7-*LifeAct-mTurquoise2*, giving rise to pK2GW7-*pPvSYMRK*::*PvSYMRK-EGFP*-p35S::*LifeAct-mTurquoise2*.

### Site-directed mutagenesis

Mutant versions of *PvSYMRK-EGFP* were generated using QuikChange^®^ Site-Directed Mutagenesis Kit (Stratagene), a pair of specific mutagenic primers (Table S1) and pENTR-MauBI_p*PvSYMRK*::*PvSYMRK-EGFP*-T35S_SacI, as template. The mutant versions were cloned in the vector pK2GW7-*LifeAct-mTurquoise2*, as described above.

### Whole-mount protocol and pharmacological treatments

The composite plants were placed in a modified Petri dish, as described by Monroy-Morales et al. (2022). Briefly, transgenic roots were whole-mounted in the chamber/coverslip containing 2–3 ml of liquid Fåhraeus medium and covered with a transparent sheet of cellulose (sweet cellophane) plain or with 6 mm diameter holes made manually, as indicated. Untreated roots were immediately examined under the microscope. Before proceeding to treatments, roots were allowed to get adapted to these conditions for 24 h at 28 °C and 16 h/8 h photoperiod. Epidermal sections of the differentiation zone of transgenic roots were selected by confocal microscopy, and inhibitors were directly administered through a hole in the cellulose sheet. Images were acquired at 0, 10, 20, 30, 40, 50, 60 min *post-*incubation with the inhibitor. Inhibitors were used at 100 μM brefeldin A (BFA), 50 μM cycloheximide (CHX), 20 μM ikarugamycin (IKA), 50 μM tyrphostin A23 (TyrA23) and 50 μM tyrphostin 51 (Tyr51).

### Confocal fluorescence imaging

Images were captured as multiple Z-planes, each one with 0.70–0.77 μm in thickness and a laser exposure time of 300–400 ms using a 3I Marianas Confocal Spinning Disk Microscope (Zeiss Observer Z.1 inverted type; Intelligent Imaging Innovations Ltd, UK), with either a water- or oil-immersion × 40 objectives (0.75 and 1.3 N.A., respectively) with an Andor Ixon 3 EMCCD camera, model DU-897E-CS0-#BV (Andor Technology, Belfast, UK) controlled by SlideBook6 (Digital Microscopy Software; Intelligent Imaging Innovations Ltd, London, UK). EGFP and mTurquoise2 were excited at 488 and 445 nm, respectively, and fluorescence emission was collected at 507 nm for EGFP and 474 nm for mTurquoise2.

### Quantitative evaluation of puncta in nonhair-epidermal cells and image processing

Quantification of fluorescent puncta in confocal fluorescence images was performed using original files without any processing or editing. From each set of confocal image stacks, three independent cells co-expressing *Pv*SYMRK-EGFP and LifeAct-mTurquoise2 were segmented and the corresponding stacks were transformed to a 2D image using the Z-project tool of ImageJ software (https://imagej.nih.gov/ij/index.html). Further the perimeter of the cell was delimited with the Straight tool and the fluorescent intracellular puncta (minute rounded dots) were quantified using Find Maxima tool. The area of each segmented cell was estimated using the Polygon Selection tool and values considered as the cell area. To facilitate the visualization of fluorescent puncta in images presented in figures, representative images were processed using deconvolution in 3D spatial dimensions performed with the Parallel Iterative Deconvolution plugin of ImageJ, using the WLP method and Wiener Filter gamma with the following parameters: weiner = 0.001, maximum number of iterations = 100, terminate = 0.01 (if mean delta is less than this value) and precision = double. This methodology requires a normalized point spread function (PSF), which was created with the Diffraction PSF 3D plugin of ImageJ using the following parameters: index of refraction of the mounting media = 1.3 or 1.5 and NA = 0.75 or 1.3, when using water-or oil-immersion objectives, respectively; wavelength = 507 nm for EGFP, 474 nm for mTurquoise2; width = 64 pixels and height = 64 pixels. Deconvolved images were edited with basic tool of ImageJ adjusting brightness and contrast and adding *pseudo*-color.

### GUS activity

Hairy roots bearing the cassette p*PvSYMRK*::*GUS-GFP* were dissected and GUS activity was detected, as described Monroy-Morales et al (2022). Briefly, roots were immersed successively for 5 min at room temperature in washing solution I (50 mM sodium phosphate buffer, pH 7.2, and 0.5 mM potassium ferrocyanide), washing solution II (50 mM sodium phosphate buffer, pH 7.2, 0.5 mM potassium ferricyanide, 2 mM EDTA-Na_2_ and 0.1% Triton X 100) and substrate solution (50 mM sodium phosphate buffer, pH 7.2, 0.5 mM potassium ferricyanide, 2 mM EDTA-Na_2_, 0.1% Triton X 100 and 0.5 mM 3-bromo-4-chloro-3-indolyl-β-D-glucuronide) at 37 °C until a blue precipitate appeared (usually 30–60 min). Roots were cleared in 1% commercial chlorine for 16 h and mounted in 30% glycerol-2% DMSO. Images were captured in a stereomicroscope SZX7 (Olympus) coupled to a QImaging MicroPublisher Color RTV-5.0 CCD Camera controlled by Image ProPlus 7.0 software.

### Statistical analyses

To validate the quantitative analysis and confirm the reproducibility of the results, statistical analysis of data from series of biological replicates were performed using GraphPad Prism version 6. The difference between values from incubation times in time-course experiments were evaluated using One-way ANOVA and Tukey’s multiple comparisons test or Friedman test, as indicated. *P* values were indicated in figure legends.

## Results

### Spatio-temporal analysis of PvSYMRK promoter activity in *Phaseolus vulgaris* transgenic roots

According to several reports, full complementation of *symrk* mutants is only achieved when *SYMRK* expression is driven by the endogenous promoter (Limpens et al. 2005; Markmann et al. 2008; Riely et al. 2013). Thus, we first analyzed the cell-specific activity of the 1622 bp fragment upstream *PvSMYRK* start codon, here referred the promoter of *PvSYMRK* (p*PvSYMRK*). As shown in Fig. S2, in uninoculated *P. vulgaris* transgenic roots, p*PvSYMRK* was active in tip-growing and mature root hair cells and nonhair-epidermal cells (Fig. S2a–e). The promoter activity was also detected in pericycle cells of the root differentiation zone and during the development of a lateral root primordium (Fig. S2a–c). In rhizobia-inoculated roots, p*PvSYMRK* activity was observed in curled root hair cells and proliferating cells underneath the infection site (Fig. S2f), as well as in those forming a nodule primordium (Fig. S2c, d), which is consistent with the activity of *MtDMI2* promoter (Bersoult et al. 2005; Riely et al 2013). In the mature nodule, the activity of p*PvSYMRK* was also distinguished in uninfected cells in the central tissue and the vascular bundles (Fig. S2e–i). A similar spatio-temporal pattern was reported in a previous study on the immunolocalization of *Pv*SYMRK in *P. vulgaris* nodulation (Sánchez-López et al. 2011).

### Redistribution of PvSYMRK-EGFP in *Phaseolus vulgaris* root hair cells responsive to rhizobia-inoculation

Before assessing the effect of rhizobia-inoculation on the subcellular distribution of *Pv*SYMRK-EGFP in *P. vulgaris* root hair cells, we first established a protocol to distinguish root hairs that are responding to rhizobia (hereafter responsive root hairs) from non-responsive root hair cells, based on the typical actin-cytoskeleton rearrangements observed in the root hair in response to Nod factors treatment or rhizobia inoculation (Sieberer and Emons 2000; Timmers 2008; Yokota et al. 2009). We took advantage of the actin-binding properties of the fluorescent F-actin reporter LifeAct-mTurquoise2, co-expressed with *Pv*SYMRK-EGFP in the transgenic roots. Non-responsive root hair cells looked the same as root hair cells from uninoculated roots, meaning they had the typical cytoplasmic streaming in the subapical region of the root hair. They also presented fine bundles of actin filaments labelled with LifeAct-mTurquoise2, that extended along the root hair and reached the apical tip (Fig. S3), as previously described (Sieberer and Emons 2000; Timmers 2008; Yokota et al. 2009). Whereas in inoculated roots, root hair cells responsive to *R. etli* CE3 pMP604 were identified by the swelling of the root hair tip and the apical accumulation of LifeAct-mTurquoise2 signal that resembles the typical accumulation of fragmented actin filaments (actin-cytoskeleton rearrangements) at the apical/subapical zone of the root hair tip in response to rhizobia and Nod factors treatment (Fig. S3; Timmers 2008; Yokota et al. 2009). Both changes precede SYMRK activation and downstream signaling, as previously described (Endre et al. 2002; Stracke et al. 2002; Kosuta et al. 2011).

**Fig. 1:**
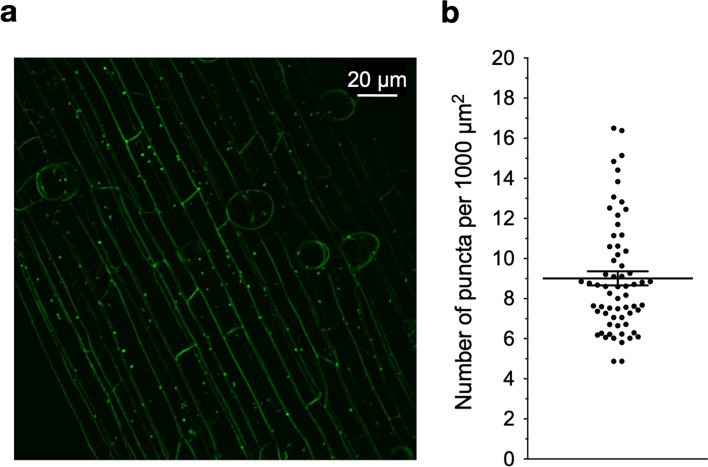
Quantitative evaluation of the puncta containing *Pv*SYMRK-EGFP in nonhair-epidermal cells from *Phaseolus vulgaris* transgenic roots. **a** Number of fluorescent puncta was assessed in 2D images of cells from uninoculated *P. vulgaris* transgenic roots expressing *Pv*SYMRK-EGFP*.* **b** Number of puncta are normalized per 1000 µm^2^ of nonhair-epidermal cell area*.* Mean value = 9 ± 0.3 SE, *n* = 64 cells from 29 independent transgenic roots. Bars correspond to mean value ± SE

Regarding its subcellular distribution in uninoculated root hair cells, *Pv*SYMRK-EGFP fluorescent signal was detected associated with the root hair PM in a discrete dotted pattern that seems homogeneously distributed (Fig. S3), as it has been reported for *Mt*DMI2 (Riely et al. 2013). Occasionally, one or two *Pv*SYMRK-EGFP labeled puncta were observed in the root hair cytoplasm. In contrast, in responsive root hair cells from *R. etli* CE3 pMP604-inoculated roots, *Pv*SYMRK-EGFP fluorescent signal disappeared from the root hair PM, and a significantly higher number of PvSYMRK-EGFP-containing puncta was observed (Fig. S3). We confirmed that non-responsive root hair cells expressing *Pv*SYMRK-EGFP, adjacent to the assessed responsive root hair cells, did not present more than one fluorescent puncta (Fig. S3). Our results were consistent with the inducing effect of Nod factors on the distribution of *Mt*DMI2-GFP at the apical PM, and the presence of fluorescent dot-like structures described as “cytoplasmic organelles” in *M. truncatula* root hairs (Riely et al. 2013). In nonhair-epidermal cells, next to growing root hair cells in uninoculated roots, *Pv*SYMRK-EGFP is also associated with the PM and intracellular puncta (Fig. 1), although fluorescent puncta are notoriously more abundant than in root hairs. Collectively, our data provide strong evidence that points to an active redistribution of *Pv*SYMRK-EGFP from the PM to intracellular puncta in *P. vulgaris* root epidermal cells and opens the possibility that abundance of *Pv*SYMRK at the PM of root hairs responsive to rhizobia could be mediated by endocytosis, as it has been described for other plant receptors (Robatzek et al. 2006; Geldner et al. 2007).

### Pharmacological treatment affects the abundance of PvSYMRK-EGFP labeled puncta in nonhair-epidermal cells

To reinforce such a hypothesis, we assessed the effect of specific inhibitors on the density of *Pv*SYMRK-EGFP-containing puncta in nonhair-epidermal cells, as they are less sensitive to mechanical actions than root hair cells (Esseling et al. 2004). As a first step, we determined that these cells have an average of 9.0 (± 0.3 SE) fluorescent puncta per 1000 µm^2^ (Fig. 1). Therefore, nonhair-epidermal cells are a suitable model for performing a comparative analysis of the density of *Pv*SYMRK-EGFP-containing puncta.

**Fig. 2:**
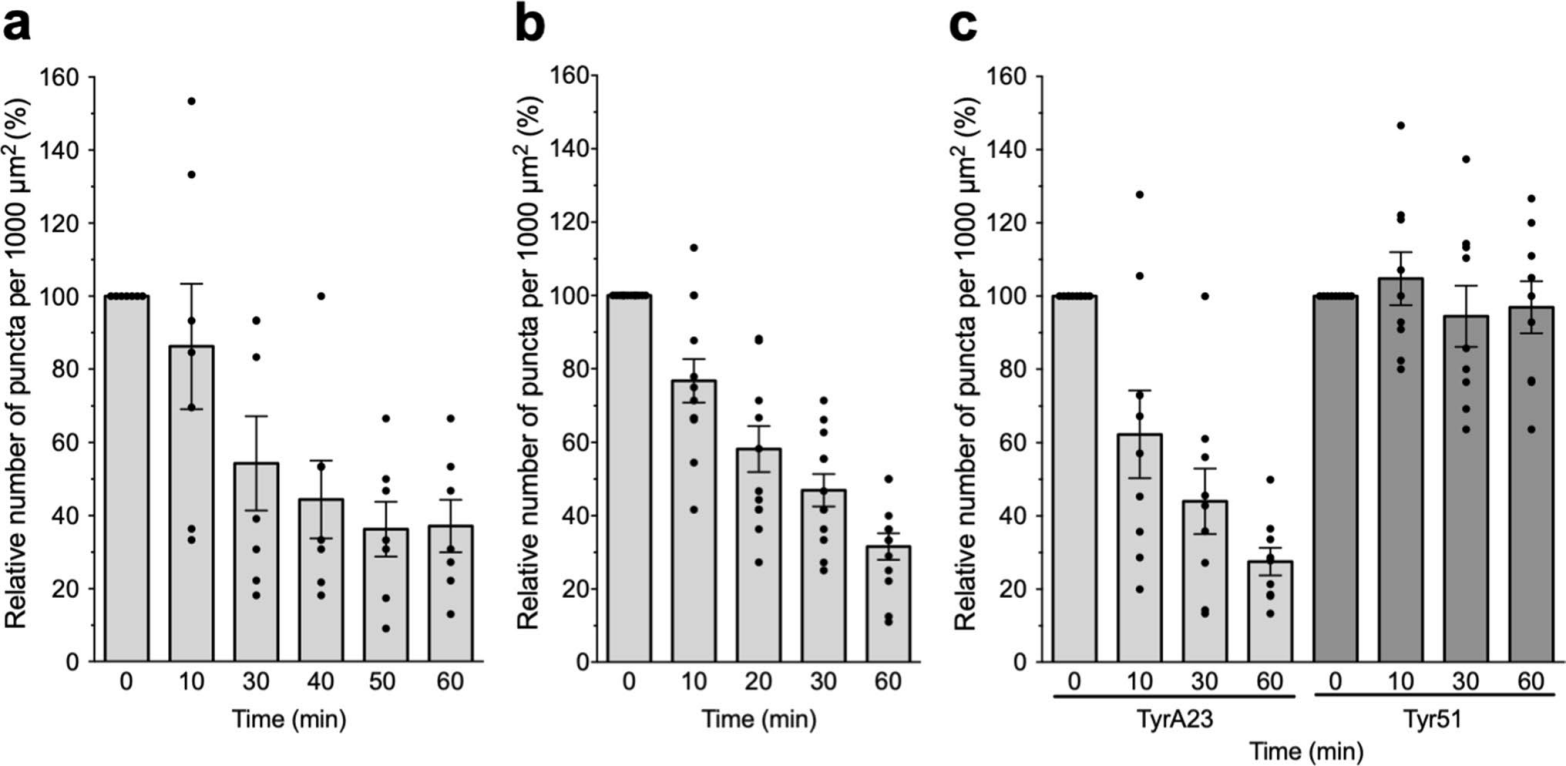
The density of puncta containing *Pv*SYMRK-EGFP is reduced in nonhair-epidermal cells treated with the protein synthesis inhibitor cycloheximide, CHX (**a**) or the endocytosis inhibitors ikarugamycin, IKA (**b**) and tyrphostin A23, TyrA23 (**c**). Tyrphostin 51 (Tyr51), a structural analog of TyrA23 that does not interfere with endocytosis, was used as a negative control (**c**)*.* Treatments were performed using 50 μM CHX, 20 μM IKA, 50 μM TyrA23 or 50 μM Tyr51. Time-lapse confocal images from *Phaseolus vulgaris* transgenic root expressing *Pv*SYMRK*-*EGFP were captured at the indicated time points. The effect of each inhibitor in individual nonhair-epidermal cells is plotted as the percentage (%) of the relative number of puncta per 1000 µm^2^ at each time point (*t*), respect to *t* = 0 (100%). Mean values of the number of puncta per 1000 µm.^2^ of cell area (± SE) at *t* = 0 are as follows: CHX, 7.72 ± 0.68, *n* = 7; IKA, 7.74 ± 0.69, *n* = 12; TyrA23, 7.94 ± 0.48, *n* = 10; Tyr51, 12.28 ± 0.99 *n* = 9. Data are from three to four plants from independent experiments. Bars indicate mean values ± SE. *n* = number of root hairs analyzed. One-way ANOVA analysis of variance on ranks and multiple comparisons (Tukey’s method) showed statistical difference at *P* < 0.001, except for data from Tyr51 treatment (*P* = 0.37). Friedman test analysis confirmed that differences in data from TyrA23 and Tyr51 treatments are statistically significant (*P* < 0.0001)

In nonhair-epidermal cells treated with the inhibitor of protein synthesis cycloheximide (CHX), a significant reduction in the percentage of fluorescent puncta was observed after 30–40 min of treatment (54 and 44%, respectively). The lowest relative value (36%) was reached at the 50–60 min time points (Fig. 2a). Hence, two fluorescent puncta populations appear to be present in nonhair-epidermal cells: a large CHX-sensitive group, involved in the transport of newly synthesized *Pv*SYMRK-EGFP, and a small group of CHX-resistant puncta, that may correspond to puncta enriched in *Pv*SYMRK-EGFP, which may transit between the PM and the TGN/EE (*trans*-Golgi network/early endosome), a plant compartment that merges secretory and endocytic/recycling pathways (Lam et al. 2009).

To assess whether the intracellular *Pv*SYMRK-EGFP-labeled puncta arise from endocytosis, nonhair-epidermal cells were treated with the inhibitors IKA and tyrphostin A23 (TyrA23), specifics of clathrin-mediated endocytosis (CME). Both inhibitors display a blocking effect on the endocytic mechanism, with no interference from subcellular trafficking activity (Dhonukshe et al. 2007). Even though the use of IKA to study the endocytic activity in plant cells is not as extensive as in animal cells, it has been established that IKA blocks the maturation and/or pinching-off of clathrin-coated vesicles from the plant PM without affecting internal vesicle trafficking (Onelli et al. 2008; Elkin et al. 2016). TyrA23, an inhibitor broadly used to prove the endocytosis of PM cargoes, disrupts CME by blocking the interaction between the µ2 subunit of the endocytic sorting adaptor AP2 complex and a PM protein cargo (Kleine-Vehn et al. 2011; Beck et al. 2012; Irani et al. 2012). We found that treatment of nonhair-epidermal cells with IKA or TyrA23 led to a gradual decrease in the number of *Pv*SYMRK-EGFP-containing puncta. Approximately 40–50% reduction was reached after 20–30 min of treatment with IKA or TyrA23 (Fig. 2b, c), attaining the lowest values at the 60 min time point (32 and 27%, respectively). In contrast, no effect was observed when the nonhair-epidermal cells were treated with 50 µM Tyr51 (Fig. 2c), a structural analog of TyrA23 that is broadly used as a negative control since it does not interfere with endocytosis (Dhonukshe et al. 2007).

**Fig. 3:**
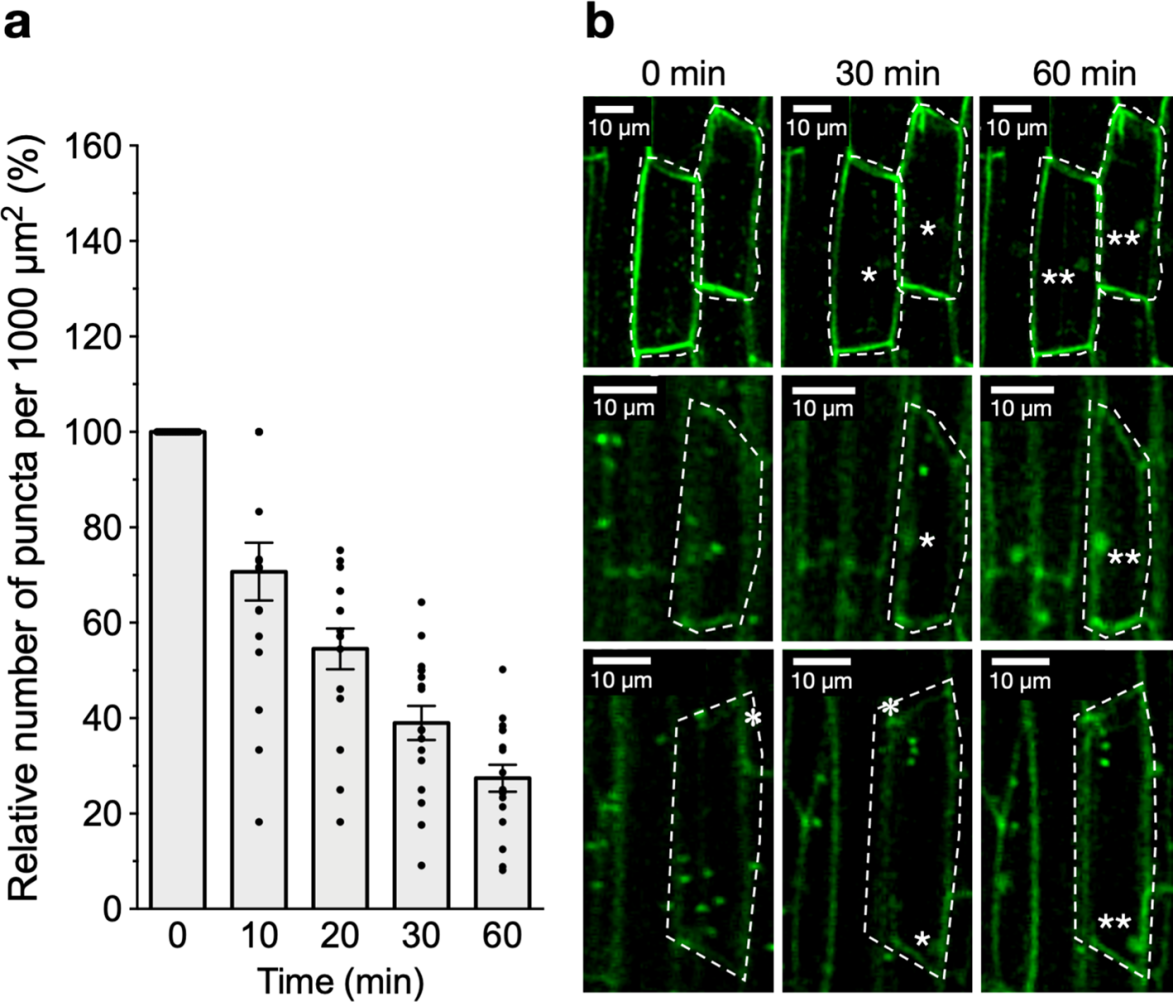
*PvSYMRK-EGFP* accumulates in brefeldin A (BFA)-induced compartments in *Phaseolus vulgaris* nonhair-epidermal root cells. Time-lapse confocal images of root epidermal cells treated with BFA were captured at the indicated time points. **a** Effect of BFA on the relative number of puncta per 1000 µm^2^ of nonhair-epidermal cell (%) was determined as indicated in Fig. 2. Mean value of the number of puncta per 1000 µm.^2^ of cell area (± SE) at t = 0 was 10.84 ± 0.93, *n* = 17 cells, from six plants from independent experiments. **b** Representative images of four independent nonhair-epidermal cells treated with BFA are showed (delimited by a dashed line). Images were captured at the indicated time points. The number of PvSYMRK-EGFP-containing puncta progressively disappeared, giving rise to BFA-induced compartments enriched in PvSYMRK-EGFP, which become apparent at time points *t* = 30 and the *t* = 60 (indicated with one or two asterisks, respectively). Bars indicate mean values ± SE. One-way ANOVA analysis of variance on ranks and multiple comparisons (Tukey’s method) showed statistical difference at *P* < 0.0001

To provide further insights into that direction, we also investigated the inhibitory effect of the fungal toxin brefeldin A (BFA). In plant cells, BFA inhibits the exocytic vesicle trafficking *post*-TGN/EE and the endosomal recycling process without affecting the endocytic activity. Thus, it became a valuable tool for investigating the endocytosis of plant receptors (Lam et al. 2009). A notable feature of BFA treatment is the coalescence of vesicles in transit between the TGN/EE and the PM, which gives rise to large subcellular structures known as BFA-induced compartments (Lam et al. 2009). *Pv*SYMRK-EGFP-containing puncta progressively disappeared in nonhair-epidermal cells treated with BFA (Fig. 3). After 20–30 min of treatment with BFA, the number of puncta was approximately halved (40–60%). At 60 min of treatment, the percentage was reduced to 27% (Fig. 3a). As expected, subcellular structures that resemble BFA-induced compartments were detected at 30–60 min time points of BFA treatment (Fig. 3b). These results are consistent with the dynamics reported in *Arabidopsis thaliana* root cells treated with this inhibitor, where BFA-induced compartments were observed after approximately 30 min of treatment (Geldner et al. 2007; Irani et al. 2012; Liu et al. 2020). As shown, the inhibitory effects of BFA, IKA and TyrA23 strongly support the notion of a constitutive CME in nonhair-epidermal cells. It is relevant to note that the PM pool of *Pv*SYMRK-EGFP was not affected by the treatment with inhibitors (Fig. S4), which may reflect a low *Pv*SYMRK-EGFP turnover.

### T589, K618 and the tetrapeptide YKTL are involved in the endocytosis of PvSYMRK-EGFP

To provide additional support to our findings, we investigated the presence of a TyrA23-targeting endocytic sorting motif YXXØ in *Pv*SYMRK. Typically, the lineal motif YXXØ is located at the cytosolic domain of transmembrane proteins that undergo endocytosis (Banbury et al. 2003; Robatzek et al. 2006; Liu et al. 2020). As a putative endocytic YXXØ motif, we identified the tetrapeptide YKTL located at the intracellular juxtamembrane region (iJXM) of SYMRK in legumes (Fig. S1). The sequence YKTL is followed by a well-conserved Ile residue and the ATP-binding site of domain I in the kinase domain (Fig. S1b). Since phospho-code of residues at or near the YXXØ motif seems to be a relevant feature in the endocytosis of plant receptors (Robatzek et al. 2006; Geldner et al. 2007; Gruszka 2013), we also identified the highly conserved T589 and T756 residues in *Pv*SYMRK, equivalent to phosphorylation sites T593 and T760 involved in the phosphorylation-dependent activation of *Lj*SYMRK (Yoshida and Parniske 2005). Those threonine residues are located at two residues from the tetrapeptide YKTL, and in the activation loop, respectively (Fig. S1). In addition, considering that phosphomimetic mutants *Lj*SYMRK K622E and *Ah*SYMRK K625E are inactive kinases (kinase-dead, KD), frequently used as negative controls in SYMRK studies (Yoshida and Parniske 2005; Samaddar et al. 2013; Saha et al. 2016; Bhattacharya et al. 2019), we also mapped the corresponding Lys residue in *Pv*SYMRK (K618; Fig. S1). To explore their structure–function relationship in *Pv*SYMRK-EGFP endocytosis, we generated mutants by individual site-directed substitution of residues T589 and T756 by Ala (non-phosphorylatable), K618 by Glu, or the deletion of the tetrapeptide YKTL. Transgenic roots expressing mutated versions of *Pv*SYMRK-EGFP were generated, except for *Pv*SYMRK(T756A)-EGFP. In this case, no transgenic roots developed even when 15 independent clones of *A. rhizogenes* carrying one of two independently constructed expression cassettes were tested.

**Fig. 4:**
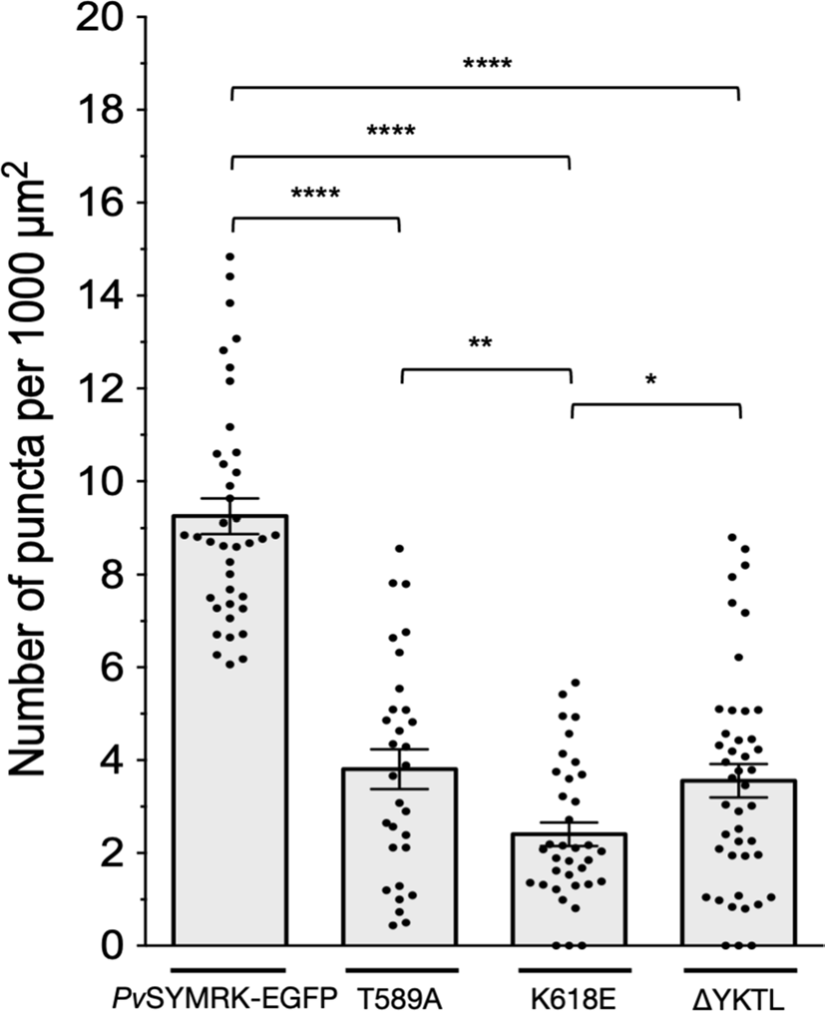
T589A, K618E and ∆YKTL mutations impair the constitutive endocytosis of *Pv*SYMRK-EGFP in *Phaseolus vulgaris* nonhair-epidermal root cells. T589 and YKTL are conserved residues present in the intracellular juxtamembrane region (iJXM) of SYMRK/DMI2 orthologues (Fig. S1). Phospho-code of T589 regulates the kinase activity of SYMRK, and tetrapeptide YKTL is an endocytic linear motif YXXØ. K618 is part of the conserved phosphotransfer VAVK motif and required for SYMRK kinase activity. Phosphomimic mutations, such as K618E inactivate the SYMRK/DMI2 kinase activity (kinase-dead, KD mutation). Positions are indicated in Fig. S1. Mean average values of the number of puncta per 1000 µm^2^ of cell area (± SE) are as follows: *Pv*SYMRK-EGFP, 9.25 ± 0.38, *n* = 39; T589A, *Pv*SYMRK(T589A)-EGFP, 3.8 ± 0.43, *n* = 30; K618E, *Pv*SYMRK(K618E)-EGFP, 2.4 ± 0.0.25, *n* = 36; ∆YKTL, *Pv*SYMRK(∆YKTL)-EGFP, 3.6 ± 0.36, *n* = 44. Values are from three to six plants from independent experiments. Bars are mean values ± SE. *n* = number of root hairs analyzed. One-way ANOVA analysis of variance on ranks and multiple comparisons (Tukey’s method) showed statistical difference at *P* < 0.0001 (****), *P* < 0.01 (**), *P* < 0.05 (*)

The analysis of transgenic roots expressing *Pv*SYMRK(T589A)-EGFP showed a significant reduction in the number of intracellular fluorescent puncta in nonhair-epidermal cells (Fig. 4). We also found a significant blockage of *Pv*SYMRK(K618E)-EGFP KD endocytosis, which is equivalent to less than 25% of the internalization activity of *Pv*SYMRK-EGFP (Fig. 4). Together, our data allow us to propose that T589-dependent phosphorylation and *Pv*SYMRK kinase activity are positive regulators of *Pv*SYMRK endocytosis.

Additionally, we observed that endocytosis of *Pv*SYMRK(∆YKTL)-EGFP was also drastically diminished to 44% (Fig. 4), which provides strong evidence that the tetrapeptide YKTL is a functional endocytic sorting motif and strengthens our data on the inhibitory effect of TyrA23 on the endocytosis of *Pv*SYMRK-EGFP. Our conclusion is consistent with studies on the functional characterization of YXXØ motifs present in cytosolic segments of several plant RLKs, RLPs (LRR receptor-like proteins), PRRs (pattern-responsive receptor), and auxin- and mineral nutrient transporters (Zipfel and Oldroyd 2017).

### *Rhizobium etli*-induced endocytosis of PvSYMRK-EGFP in *Phaseolus vulgaris* root hair cells is Nod factors-dependent and requires the YXXØ motif and an active kinase

**Fig. 5:**
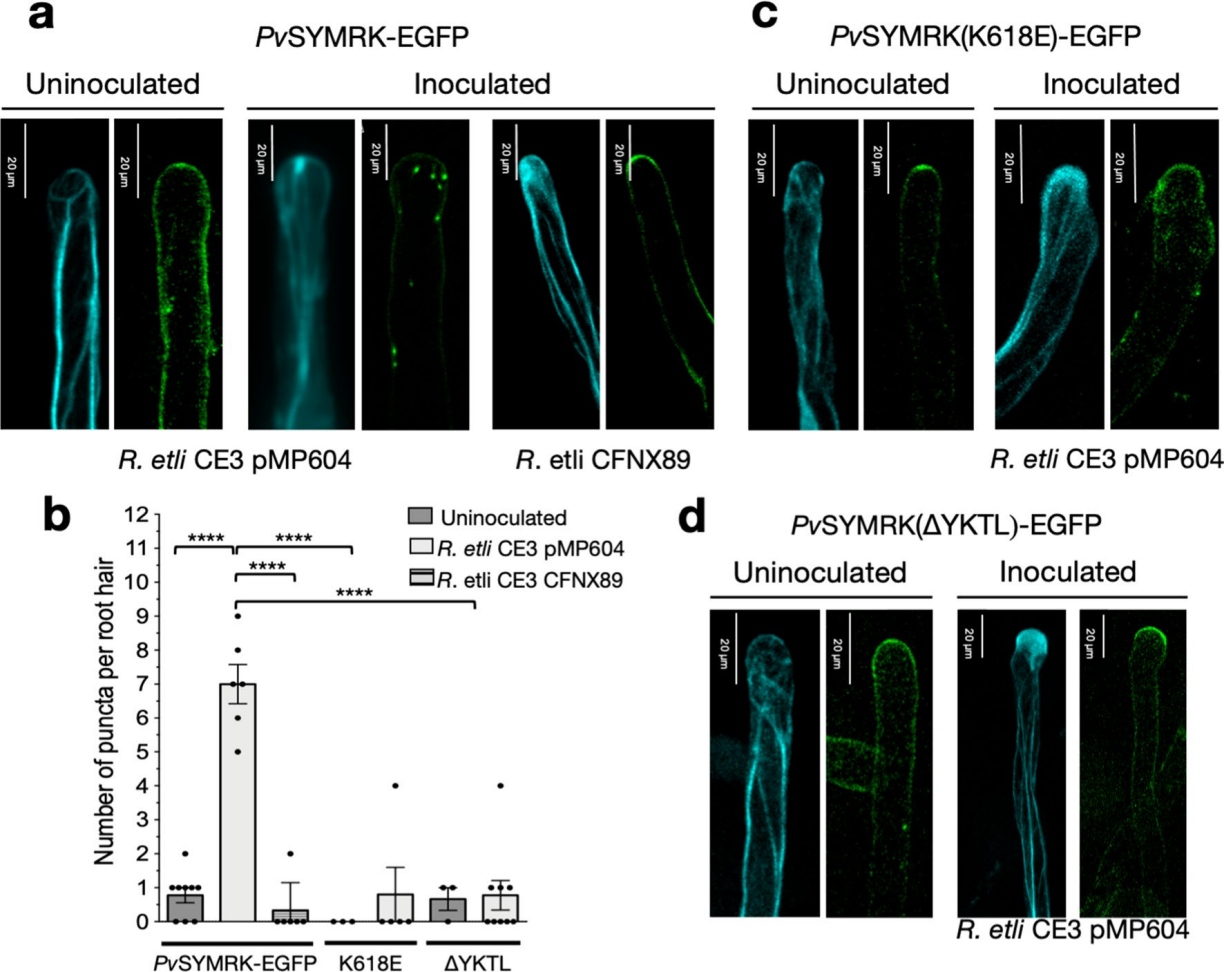
*Pv*SYMRK-EGFP endocytosis is induced in rhizobia-responsive root hair cells from *Phaseolus vulgaris* transgenic roots and it depends on Nod factors, the YXXØ motif and a fully-active kinase. **A**–**d** In *P. vulgaris* roots inoculated with either *Rhizobium etli* CE3 pMP604 (a Nod factors *plus* strain) or *R. etli* CE3 CFNX89 (a Nod factors minus strainBrom et al. 1992; Corvera et al. 1999) (3dpi), responsive root hair cells are distinguished from the non-responsive root hair cells by the accumulation of LifeAct-mTurquoise2 signal (in cyan) at the apical zone, as described in Fig. 2. In root hair cells from uninoculated roots, unmutated and mutated *Pv*SYMRK-EGFP (all in green) are mainly associated with the apical PM and eventually one or two intracellular puncta are observed (**a–d**). In responsive-root hair cells from inoculated roots with *R. etli* CE3 pMP604, a higher number of unmutated *Pv*SYMRK-EGFP-labeled puncta are found (**a**, **b**), whereas no significant effect is observed in root hairs from roots inoculated with the rhizobia Nod factors minus strain (CFNX89) (**a**, **b**), indicating that rhizobia-induced endocytosis of *Pv*SYMRK-EGFP depends on the presence of Nod factors. Neither *Pv*SYMRK(K618E)-EGFP nor *Pv*SYMRK(ΔYKTL)-EGFP undergo endocytosis in responsive-root hairs from roots inoculated with *R. etli* CE3 pMP604 (**b–d**). The average in number of puncta ± SE rhizobia-responsive root hair cells, are as follows: unmutated *Pv*SYMRK-EGFP from roots uninoculated or inoculated with *R. etli* CE3 pMP604 or *R. etli* CE3 CFNX89), 0.78 ± 0.22, *n* = 9, 7 ± 0.6, *n* = 6 and 0.33 ± 0.3, *n* = 6, respectively; *Pv*SYMRK(K618E)-EGFP, 0 puncta, *n* = 3 and 0.8 ± 0.8, *n* = 5, respectively; *Pv*SYMRK(ΔYKTL)-EGFP, 0.67 ± 0.33,* n* = 3 and 0.78 ± 0.43, respectively. Values and bars are mean values ± SE, *n* = number of root hairs cells analyzed. One-way ANOVA analysis of variance on ranks and multiple comparisons showed statistical difference (Tukey’s method) at *P* < 0.0001 (****)

To better characterize the rhizobia-induced endocytic activity of *Pv*SYMRK-EGFP observed in Fig. S3, we analyzed the subcellular distribution in root hairs from roots inoculated with *R. etli* CFNX89, a rhizobia strain that does not produce Nod factors (Brom et al. 1992; Corvera et al. 1999). We found that *R. etli* CFNX89 inoculation did not induce a redistribution of *Pv*SYMRK-EGFP, it remains mainly associated with the root hair PM, suggesting that Nod factors are involved in the induced endocytosis of *Pv*SYMRK-EGFP (Fig. 5a, b). We also observed that root hairs presented a rearranged actin-cytoskeleton (Fig. 5a), an unexpected result considering that such rearrangement typically occurs in response to exposure to exogenous Nod factors or with rhizobia strains competent in the synthesis and secretion of active Nod factors (Sieberer and Emmons 2000; Timmers 2008; Yakota et al. 2009; Liang et al. 2021). Interestingly, actin-related proteins are differentially expressed in roots inoculated with wild-type rhizobia and an exopolysaccharide-deficient (*exoY*) rhizobia mutant (Jones et al. 2008).

To functionally test the role of YKTL and K618 in the induced endocytic activity of *Pv*SYMRK-EGFP, we performed a quantitative analysis of the subcellular distribution of unmutated *Pv*SYMRK(∆YKTL)-EGFP and *Pv*SYMRK(K618E)-EGFP KD in root hair cells in the context of uninoculated and *R. etli* CE3 pMP604-inoculated *P. vulgaris* transgenic roots. Consistently, only unmutated *Pv*SYMRK-EGFP undergoes induced endocytosis in root hair cells actively responding to *R. etli* CE3 pMP604 (Fig. 5). Deletion of the tetrapeptide YKTL and K618E mutation entailed a detrimental effect on the induced endocytic activity of *Pv*SYMRK-EGFP in root hair cells (Fig. 5).

## Discussion

SYMRK/DMI2/NORK is a root-specific RLK that plays an essential role in plant–microbe symbiotic interactions (Markmann et al. 2008). Collective data from molecular, biochemical, genetics and microscopical analysis revealed that activation of SYMRK triggers a signal transduction cascade that regulates an extensive signaling network associated with infection and nodule organogenesis (Yang et al. 2022). However, despite its relevant functions in nodulation, the cellular processes linking SYMRK activation to downstream cellular mechanisms underlying SYMRK signaling remain to be uncovered. The key questions are what is the identity of the extracellular elicitor(s) or ligand that activates SYMRK and what is the functional connection between intracellular SYMRK interactors, such as *LjSIE3*, *LjSINA4* and *MtPUB* (Den Herder et al. 2012; Liu et al. 2018), among others, and SYMRK activation. The identification of suppressor mutants of the SYMRK-deficient phenotype would also be a valuable tool.

To provide insights into the cellular mechanisms involved in SYMRK regulation, we applied a different approach, the visualization of the subcellular dynamics of SYMRK in the root hair in response to rhizobia. Since root hair phenotype of *M. truncatula* and *L. japonicus dmi2/symrk* mutants have not been fully addressed, we performed our analysis in *P. vulgaris* transgenic roots expressing the cassette p*PvSYMRK::PvSYMRK-EGFP* in a wild-type background. We did not find a significant difference in the expression of *PvSYMRK* and other common symbiotic signaling pathway (CSSP) genes in the transgenic roots respect to *P. vulgaris* wild-type roots. We demonstrated that rhizobia induce *Pv*SYMRK-EGFP endocytosis in *P. vulgaris* root hair cells. Whereas in epidermal cells from uninoculated roots, the endocytosis of *Pv*SYMRK-EGFP is constitutive. In addition, we provide insights into the role played by the T589 phospho-code, the K618-dependent kinase activity, and the linear motif YKTL, as positive regulators of the endocytic activity of this receptor.

We first focused on describing the subcellular distribution of *Pv*SYMRK in *P. vulgaris* root epidermal cells. We found that *Pv*SYMRK-EGFP associates with the apical PM and intracellular puncta in root hair cells and nonhair-epidermal cells (Fig. 1 and S3). We also observed that inoculation with rhizobia induce a redistribution of *Pv*SYMRK-EGFP in responsive root hair cells, meaning it disappears from the PM and intracellular puncta become more abundant in root hairs that present fragmented actin filaments in the apical zone (Fig. S3). *Mt*DMI2-GFP presented a similar pattern in *M. truncatula* root hair cells treated with Nod factors (Riely et al. 2013), suggesting that clearance of SYMRK from the apical PM of rhizobia-responsive root hair cells is a cellular mechanism that contributes to the homeostasis of this receptor at early stages of epidermal infection in legume:rhizobia nodulation. It is tempting to hypothesize that endocytosis SYMRK is a cellular mechanism common in plant symbiosis.

The functional relationship between a dual distribution of plant PM-associated RLKs and endocytosis was demonstrated by studying the activity of BRI1, FLS2, BOR1 and PIN proteins. Further studies led to the identification of coreceptors, inhibitory proteins and other regulators associated with plant receptor endocytosis (Robatzek et al. 2006; Dhonukshe et al. 2007; Geldner et al. 2007; Kleine-Vehn et al. 2011; Beck et al. 2012; Irani et al. 2012; Liu et al. 2020).

To support the hypothesis on the endocytic activity of *Pv*SYMRK-EGFP, we considered it imperative to document the effect of endocytosis inhibitors on *Pv*SYMRK-EGFP subcellular distribution, as well as the role played by specific residues present in *Pv*SYMRK. We found that the treatment with IKA or TyrA23, disruptors of CME (Onelli et al. 2008; Elkin et al. 2016), and the deletion of the tetrapeptide YXXØ (*Pv*SYMRK(∆YKTL)-EGFP; Figs. 2, 4 and 5) have a detrimental effect on the number of intracellular puncta bearing *Pv*SYMRK-EGFP, which indicate that *Pv*SYMRK-EGFP undergoes CME. The motif YXXØ is the target of TyrA23 and the binding site for the µ2 subunit of the endocytic sorting AP2 complex of CME (Kadlecova et al. 2017). The driving force of the YXXØ motif in CME was originally described in mammalian cells (Banbury et al. 2003). Soon, it became evident that it has a similar function in plant CME. Site-directed mutations of YXXØ impair CME of *Le*Eix2 (Bar and Avni 2009), *Os*NAS2 (rice PM nicotianamine synthase; Nozoye et al. 2014) and BRI1 (Liu et al. 2020). Moreover, the motif YXXØ has been associated with events that connect endocytosis and polar localization of BOR1 (Takano et al. 2010) and PIN proteins (Glanc et al. 2018).

We also showed that in brefeldin A (BFA)-treated cells, *Pv*SYMRK-EGFP accumulates in subcellular structures that resemble BFA-induced compartments (Fig. 3). Therefore, homeostasis of *Pv*SYMRK-EGFP at the PM of epidermal cells may involve an endosomal-recycling step, as it is the case of BRI1, FLS2, BOR1, PIN1/PIN2, and FERONIA (Robatzek et al. 2006; Geldner et al. 2007; Dhonukshe et al. 2007; Lam et al. 2009; Kleine-Vehn et al. 2011; Beck et al. 2012; Irani et al. 2012; Liu et al. 2020).

Additionally, we found that non-phosphorylatable T589A mutation compromises the endocytosis of *Pv*SYMRK-EGFP (Fig. 4). T593, equivalent to *Pv*SYMRK T589, is critical for the phosphorylation-dependent activation of *Lj*SYMRK (Yoshida and Parniske 2005). Therefore, it is plausible that efficient endocytosis of *Pv*SYMRK-EGFP depends on the phospho-code of T589. The phosphorylation status of specific Thr or Ser residues, located at the intracellular juxtamembrane region (iJXM) region, is relevant for the endocytosis of the boron importer *At*NIP5;1 (Thr in the TPG repeats; Takano et al. 2010; Bertoni 2017) and the metal transporter *At*NRAMP (Ser20; Castaings et al. 2021). Whether the phospho-code of BRI T842 and T872 (Wang et al. 2005; Oh et al. 2012), FLS2 T867 (Robatzek et al. 2006) and XA21 T680 and T705 (Chen et al. 2010) are related to their endocytic activity is an issue that remains to be explored.

Interestingly, kinase-dead mutation K618E prevents *Pv*SYMRK-EGFP endocytosis (Figs. 4 and 5), which denotes a functional relationship between a fully active receptor and endocytosis. Although such a relationship has not been directly addressed in other plant RLKs that undergo endocytosis, the inhibitory effect of kinase-dead mutants on the corresponding signaling pathway offers insights to be considered. For instance, using BRI1(K911E) KD, it became evident that BRI1 kinase activity is crucial for BRI1 ubiquitination, a critical step in BRI1 endocytosis (Liu et al. 2020). Moreover, functional interaction between BRI1 and its coreceptor BAK1 is defective in plants expressing either BRI1 KD or BAK1 KD (Wang et al. 2005). In contrast, BAK1 kinase activity is not required for flg22-induced formation of a FLS2-BAK1 KD complex, but it blocks the activation of downstream signaling (Schulze et al. 2010).

Currently, induced endocytosis of a PM-associated RLK is described as a cellular process involved in a specific step of molecular communication between the outside and the inside of the cell. In this case, endocytosis is usually induced by the binding of an RLK-specific external ligand or elicitor, which triggers either the activation or the inhibition of the respective RLK-dependent downstream signaling pathway (Claus et al. 2018). It is, therefore, reasonable to postulate that rhizobia-induced endocytosis of SYMRK modulates the duration and amplitude of the SYMRK-dependent signaling pathway, essential for the epidermal infection. In that regard, identification of the molecular signals that trigger this endocytic step is crucial. Our results provide some clues in that direction. We found that the rhizobia-induced endocytosis of PvSYMRK-EGFP appears to be dependent on Nod factors, as it is not induced in root hairs responsive a Nod factors-deficient strain (*R*. *etli* CFNX89, Fig. 5). An unexpected result was the actin-cytoskeleton rearrangements induced in root hairs *R*. *etli* CFNX89, meaning in absence of Nod factors, which contrast with our current understanding that indicates that such rearrangements are in response to the exposure of exogenous Nod factors (Sieberer and Emmons 2000; Timmers 2008; Yakota et al. 2009; Liang et al. 2021). Notwithstanding the specific response to Nod factors treatment, the possibility that an unknown bioactive signal, different from Nod factors may also be involved in the induced cytoskeleton rearrangements. An intriguing option that has not been explored is whether exopolysaccharides (EPS) and lipopolysaccharides (LPS) synthesized by rhizobia (Jones et al. 2008; Maillet et al. 2020; Acosta-Jurado et al. 2021) are elicitors of the actin-depolymerization activity leading to actin cytoskeleton rearrangements in response to rhizobia inoculation, as it has been found associated with plant immunity responses (Zipfel and Oldroyd 2017; Sassmann et al. 2018; Wang et al. 2022).

Undoubtedly, the availability of receptor-specific ligands has been decisive in identifying coreceptors and inhibitors, as well as in deciphering the dynamics of the induced endocytosis of BRI1, FLS2, CERK, BOR1, LeEix2, among others (Claus et al. 2018; Mao and Li 2020).

Regarding the identity of a putative SYMRK coreceptor, according to Antolín-Llovera et al. (2014b) in rhizobia-inoculated *L. japonicus* roots, *Lj*SYMRK forms a heterodimer with the Nod factors coreceptor *Lj*NFR5. Such an interaction seems to be mediated by the *Lj*SYMRK LRRs, whereas in uninoculated roots, the *Lj*SYMRK malectin-like domain impedes *Lj*SYMRK-*Lj*NFR5 interaction and promotes *Lj*SYMRK degradation (Antolín-Llovera et al. 2014b). Additionally, an unexpected functional relationship between SYMRK and BAK1, involved in rhizobial suppression of plant immune response, has recently been reported (Feng et al. 2021). BAK1/SERK3 is a versatile coreceptor that forms heterodimers with diverse RLKs, such as BRI1, FLS2, EFR, BIR1, PEPR1/PEPR2, among others (Chinchilla et al. 2009). Thus, BAK1 is a central player in processes associated with plant hormone regulation, development, programmed cell death and immune responses (Gao et al. 2019; Mao and Li 2020). On the other hand, our current understanding indicates that rhizobia signaling occurs through MAMP (microbe-associated molecular pattern) elicitors that transiently activate a plant immune response, also termed MTI (MAMP- triggered immunity), and Nod factors, essential for an optimal epidermal infection. Interestingly, it also seems that Nod factors are involved in suppressing plant immune response (Antolín-Llovera et al. 2014a; Cao et al. 2017; Yang et al. 2022). Therefore, a balance between immune response and symbiosis appears to be determinant for the setting of the initial steps of rhizobia infection. According to Feng et al. (2021), the MTI induced in *L. japonicus* roots treated with the flg22 elicitor is suppressed by a rhizobia-induced SYMRK-BAK1 interaction. Furthermore, *ljbak1*-deficient CRISPR-Cas9 mutants display a higher number of infection events than in wild-type roots, even though the nodule density in both group of plants was comparable (Feng et al. 2021). Therefore, the final balance of the crosstalk between BAK1 and SYMRK would be that BAK1, a negative regulator of rhizobial infection, is inhibited by SYMRK, allowing the infection and nodulation to take place (Feng et al. 2021).

Future challenges in the study of SYMRK will be focused on deciphering the functional and cellular relationship between SYMRK activation, rhizobia-induced endocytosis, downstream signaling and BAK1-interaction associated with the immune response and the infection processes at the initial stages of nodulation, and eventually in the nonhair-epidermal infection during mycorrhizal and actinorhizal symbiosis.

### *Author contribution statement*

RDD designed the experiments and conducted the cloning and confocal microscopy experiments and image analysis. KFC and MAJV conducted the promoter cloning and GUS staining analysis; RSL designed and supervised the study. RDD and RSL wrote the manuscript. All authors read and approved the submitted version of the manuscript.

## Supplementary Information

Below is the link to the electronic supplementary material.

Supplementary file1 Table S1 Oligonucleotides used in this study (PDF 43 KB)

Supplementary file2 Fig. S1 a Schematic representation of pPvSYMRK::PvSYMRK-EGFP. pPvSYMRK promoter sequence (1622 pb); PvSYMRK, sequence coding for a polypeptide of 919 amino acid residues, MLD, malectin-like domain; LRR, leucine-rich repeat; TMD, transmembrane domain; iJXM, intracellular juxtamembrane; NCS, non-conserved sequence; EGFP, enhanced GFP. b Partial sequence alignment of *Medicago truncatula* DMI2 (Medtr5g030920), *Lotus japonicus* SYMRK (AF492655), *Phaseolus vulgaris* SYMRK (Phvul.002G143400) and *Glycine max* SYMRK (Glyma01g02451). The sequences of the ATP binding site of domain I and the phosphotransfer motif VAVK are indicated by one and two asterisks, respectively. Residues that were substituted or deleted in this study are framed in red (TIFF 11119 KB)

Supplementary file3 Fig. S2 PvSYMRK promoter activity in uninoculated and *Rhizobium tropici* CIAT899 GUS- inoculated *Phaseolus vulgaris* roots. In uninoculated roots, the promoter is active in tip growing (a-c) and mature (d) root hair cells, and in nonhair epidermal cells (e). The promoter activity is also detected in the pericycle and dividing cells forming the domed shape of the LRP (g). At later stages, the activity is restricted to the cells at the base of the LRP (h-i). Upon rhizobial inoculation, pPvSYMRK activity is detected in root hair cells, curled in response to rhizobia and in cortical cells undergoing the initial divisions underneath the infection site (f). During the nodule organogenesis, the pPvSYMRK activity is observed in the cells forming the nodule primordium (i), as well as in a not yet well-organized central tissue and the provascular trace in the young nodule (j). In the mature nodule, the promoter is active in the vascular bundle and in uninfected cells in the central tissue (k-m). The promoter activity was revealed through histochemical staining of GUS activity in transgenic roots expressing the cassette pPvSYMRK::GFP-GUS. ccd, cortical cell division; ct, central tissue; ds, domed shape of the LRP; ic, infected cell; LRP, lateral root primordium; np, nodule primordium; pc, pericycle cells; pv, provascular trace; uic, uninfected cell; vb, vascular bundle (TIFF 6256 KB)

Supplementary file4 Fig. S3 Representative images showing the subcellular distribution of PvSYMRK-EGFP in root hair cells from *Phaseolus vulgaris *roots uninoculated and inoculated with *R. etli* CE3 pMP604. In root hair cells from uninoculated roots, PvSYMRK-EGFP is associated with the apical plasma membrane (PM) in a discrete dotted pattern. In roots inoculated with *R. etli *CE3 pMP604 (3 dpi), PvSYMRK-EGFP disappear from the PM and PvSYMRK-EGFP-labeled intracellular puncta are more abundant. Rhizobia-responsive root hair cells are easily distinguished from non-responsive by the accumulation of fragmented actin filaments at the apical/subapical zone of the root hair tip (indicated by a white square), as displayed by LifeAct-mTurquoise2 signal (TIFF 8340 KB)

Supplementary file5 Fig. S4 The PM pool of PvSYMRK-EGFP in nonhair-epidermal cells was not affected by the treatment with inhibitors. PM/intracellular relative fluorescence intensity was determined as described by Luo and Russinova (2017). Data are from representative cells treated with inhibitors cyclohexamide (CHX), ikarugamycin (IKA), tyrphostin A23 (TyrA23), tyrphostin 51 (Tyr51) and brefeldin A (BFA) as described in Fig. 2 and 3. Values and bars are mean values ± SE, n = 7 cells. One-way ANOVA analysis of variance on ranks and multiple comparisons (Tukey’s method) showed no significant statistical difference (TIFF 11119 KB)

## Data Availability

All data generated or analyzed during this study are included in this published article and its supplementary information files.

## Abbreviations

BRI1: BRASSINOSTEROID INSENSITIVE 1
CME: Clathrin-mediated endocytosis
FLS2: FLAGELLIN SENSING 2
IKA: Ikarugamycin
IT: Infection thread
KD: Kinase-dead mutant
PM: Plasma membrane
RLK: Receptor-like kinase
SYMRK: Symbiosis receptor-like kinase
