# Supplementary material for: Rhizobia induce SYMRK endocytosis in Phaseolus vulgaris root hair cells
Source: Planta. 2023 Mar 16;257(4):83. doi: 10.1007/s00425-023-04116-0 (PMC10020325; doi:10.1007/s00425-023-04116-0)
Supplement: Supplementary file 1 — Supplementary file1 Table S1 Oligonucleotides used in this study (PDF 43 KB) [file 425_2023_4116_MOESM1_ESM.pdf]

Table S1. Oligonucleotides used in this study

Primer	Sequence
pPvSYMRKH5’ UP	5’-CACCAAGCTTCCGGGCTGGTAAAATCTTAGGC-3’
pPvSYMRK3’ LW	5’-TCTAGAGCTTGCTTAATCCCATAATTCTG-3’
LifeAct LUP	5’-CACCATGGGTGTCGCAGATTTGATCAAGAAATTCGAAAGCATCTCAAAGGAAGAAGTGAGCAAGGGCGAG-3’
mTurquoise2 LW	5’-CTACTTGTACAGCTCGTCCATGCC-3’
5UTR UP	5´- CACCCCTGCCGTTTCCAGGCTTG -3´
PvSYMRK LW	5'-TCTCGGCTGTGGATGGGACAAGGC-3'
MauB1pPvSYMRK UP	5’-CACCCACCTGCAAGCTTCCGGGCTGGTAAAATCTTAGGC-3’
T35S-SacI LW	5´-AAGAGCTCCCTCTAGAGGGCCCGACG-3’
T589A UP	5’-TTTGGAAGATATAGAGGTGGCCGCAGAAAGGTACAAAACATTG-3’
T589A LW	5’-CAATGTTTTGTACCTTTCTGCGGCCACCTCTATATCTTCCAAA-3’
T756A UP	5’-TCTTGAAGTCAGAGGAGCTGCAGGGTACCTGGATCC-3’
T756A LW	5’-GGATCCAGGTACCCTGCAGCTCCTCTGACTTCAAGA-3’
K618E UP	5’-TCAAGAAGTGGCAGTGGAAGTCCGTTCAGCCA-3’
K618E LW	5’-TGGCTGAACGGACTTCCACTGCCACTTCTTGA-3’
ΔYKTL UP	5’-ATATAGAGGTGGCCACAGAAAGGATAGGGGAAGGAGGATTTGGTT-3’
ΔYKTL LW	5’-AACCAAATCCTCCTTCCCCTATCCTTTCTGTGGCCACCTCTATAT-3’
