# Supplementary figures and images for: Rhizobia induce SYMRK endocytosis in Phaseolus vulgaris root hair cells
Source: Planta. 2023 Mar 16;257(4):83. doi: 10.1007/s00425-023-04116-0 (PMC10020325; doi:10.1007/s00425-023-04116-0)

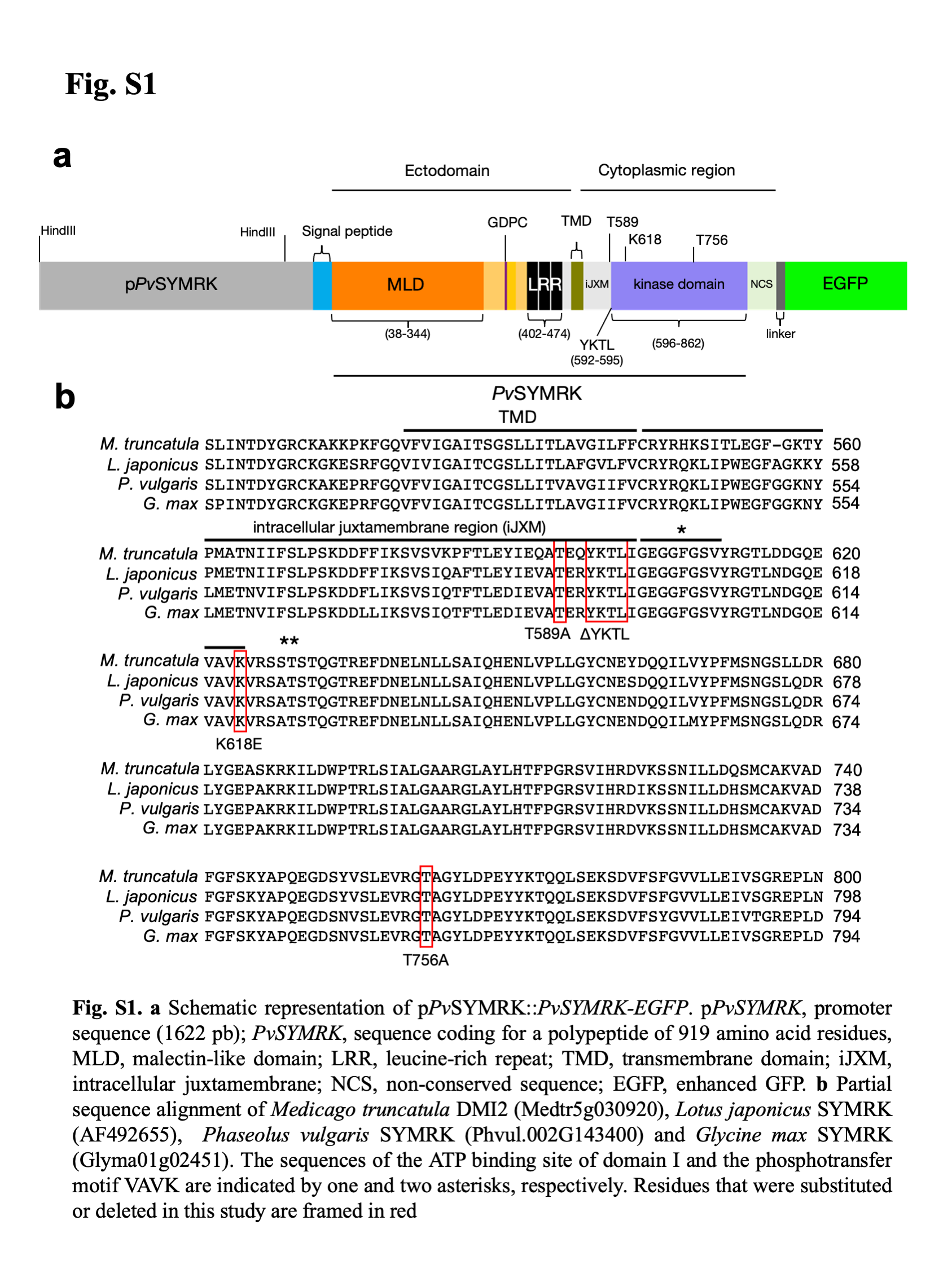

Supplement: Supplementary file 2 — Supplementary file2 Fig. S1 a Schematic representation of pPvSYMRK::PvSYMRK-EGFP. pPvSYMRK promoter sequence (1622 pb); PvSYMRK, sequence coding for a polypeptide of 919 amino acid residues, MLD, malectin-like domain; LRR, leucine-rich repeat; TMD, transmembrane domain; iJXM, intracellular juxtamembrane; NCS, non-conserved sequence; EGFP, enhanced GFP. b Partial sequence alignment of Medicago truncatula DMI2 (Medtr5g030920), Lotus japonicus SYMRK (AF492655), Phaseolus vulgaris SYMRK (Phvul.002G143400) and Glycine max SYMRK (Glyma01g02451). The sequences of the ATP binding site of domain I and the phosphotransfer motif VAVK are indicated by one and two asterisks, respectively. Residues that were substituted or deleted in this study are framed in red (TIFF 11119 KB) [file 425_2023_4116_MOESM2_ESM.tiff]

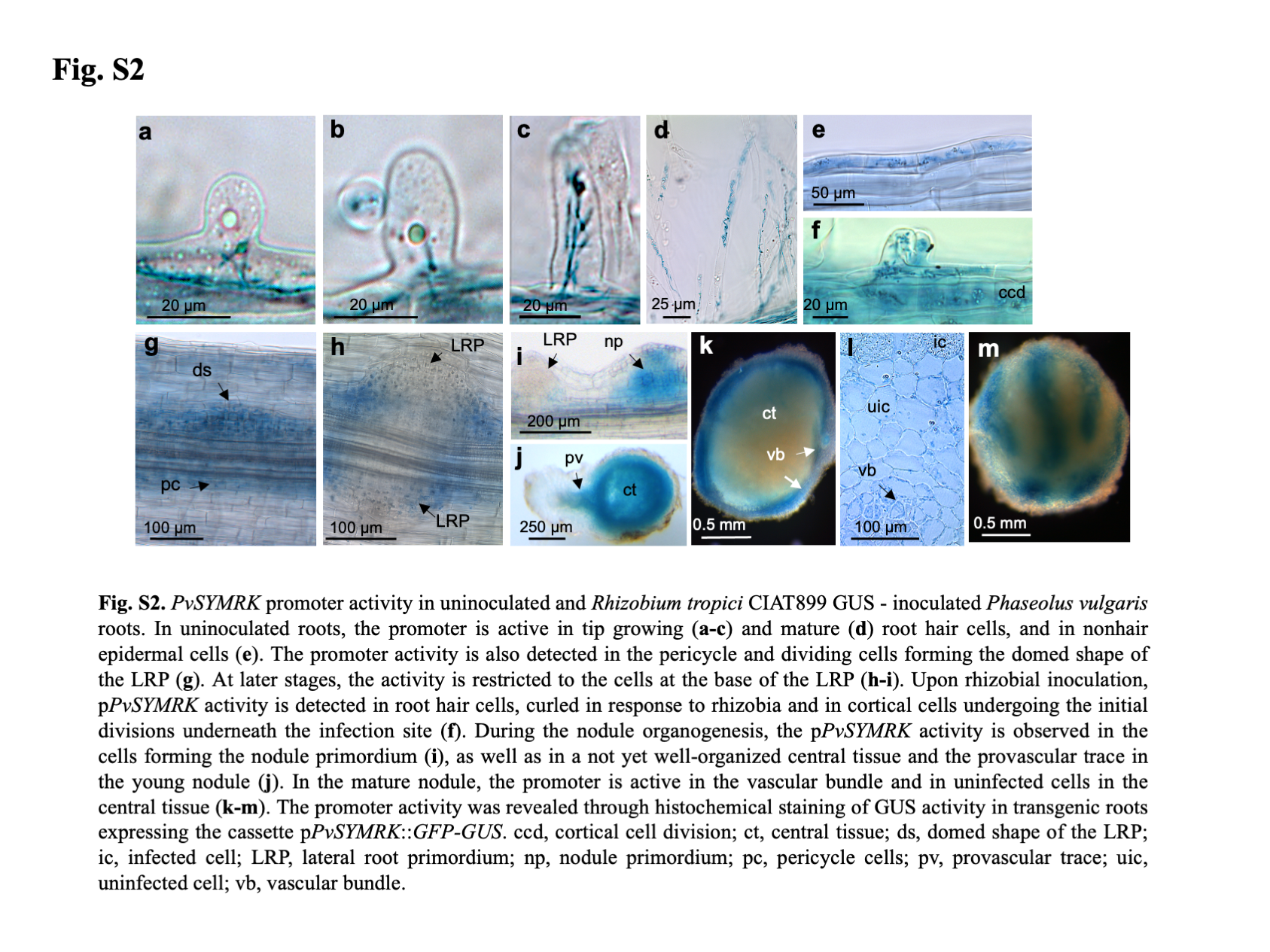

Supplement: Supplementary file 3 — Supplementary file3 Fig. S2 PvSYMRK promoter activity in uninoculated and Rhizobium tropici CIAT899 GUS- inoculated Phaseolus vulgaris roots. In uninoculated roots, the promoter is active in tip growing (a-c) and mature (d) root hair cells, and in nonhair epidermal cells (e). The promoter activity is also detected in the pericycle and dividing cells forming the domed shape of the LRP (g). At later stages, the activity is restricted to the cells at the base of the LRP (h-i). Upon rhizobial inoculation, pPvSYMRK activity is detected in root hair cells, curled in response to rhizobia and in cortical cells undergoing the initial divisions underneath the infection site (f). During the nodule organogenesis, the pPvSYMRK activity is observed in the cells forming the nodule primordium (i), as well as in a not yet well-organized central tissue and the provascular trace in the young nodule (j). In the mature nodule, the promoter is active in the vascular bundle and in uninfected cells in the central tissue (k-m). The promoter activity was revealed through histochemical staining of GUS activity in transgenic roots expressing the cassette pPvSYMRK::GFP-GUS. ccd, cortical cell division; ct, central tissue; ds, domed shape of the LRP; ic, infected cell; LRP, lateral root primordium; np, nodule primordium; pc, pericycle cells; pv, provascular trace; uic, uninfected cell; vb, vascular bundle (TIFF 6256 KB) [file 425_2023_4116_MOESM3_ESM.tiff]

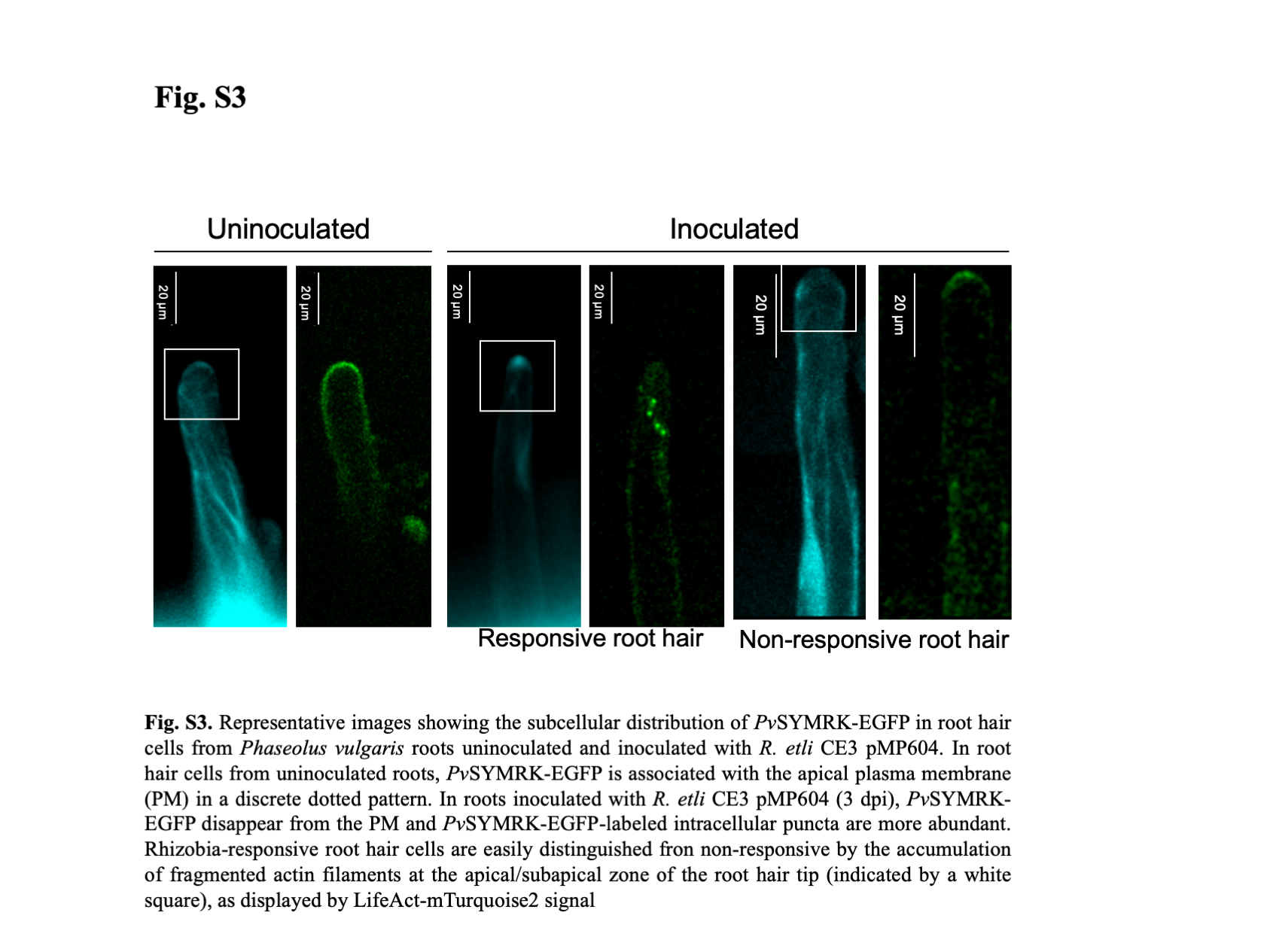

Supplement: Supplementary file 4 — Supplementary file4 Fig. S3 Representative images showing the subcellular distribution of PvSYMRK-EGFP in root hair cells from Phaseolus vulgaris roots uninoculated and inoculated with R. etli CE3 pMP604. In root hair cells from uninoculated roots, PvSYMRK-EGFP is associated with the apical plasma membrane (PM) in a discrete dotted pattern. In roots inoculated with R. etli CE3 pMP604 (3 dpi), PvSYMRK-EGFP disappear from the PM and PvSYMRK-EGFP-labeled intracellular puncta are more abundant. Rhizobia-responsive root hair cells are easily distinguished from non-responsive by the accumulation of fragmented actin filaments at the apical/subapical zone of the root hair tip (indicated by a white square), as displayed by LifeAct-mTurquoise2 signal (TIFF 8340 KB) [file 425_2023_4116_MOESM4_ESM.tiff]

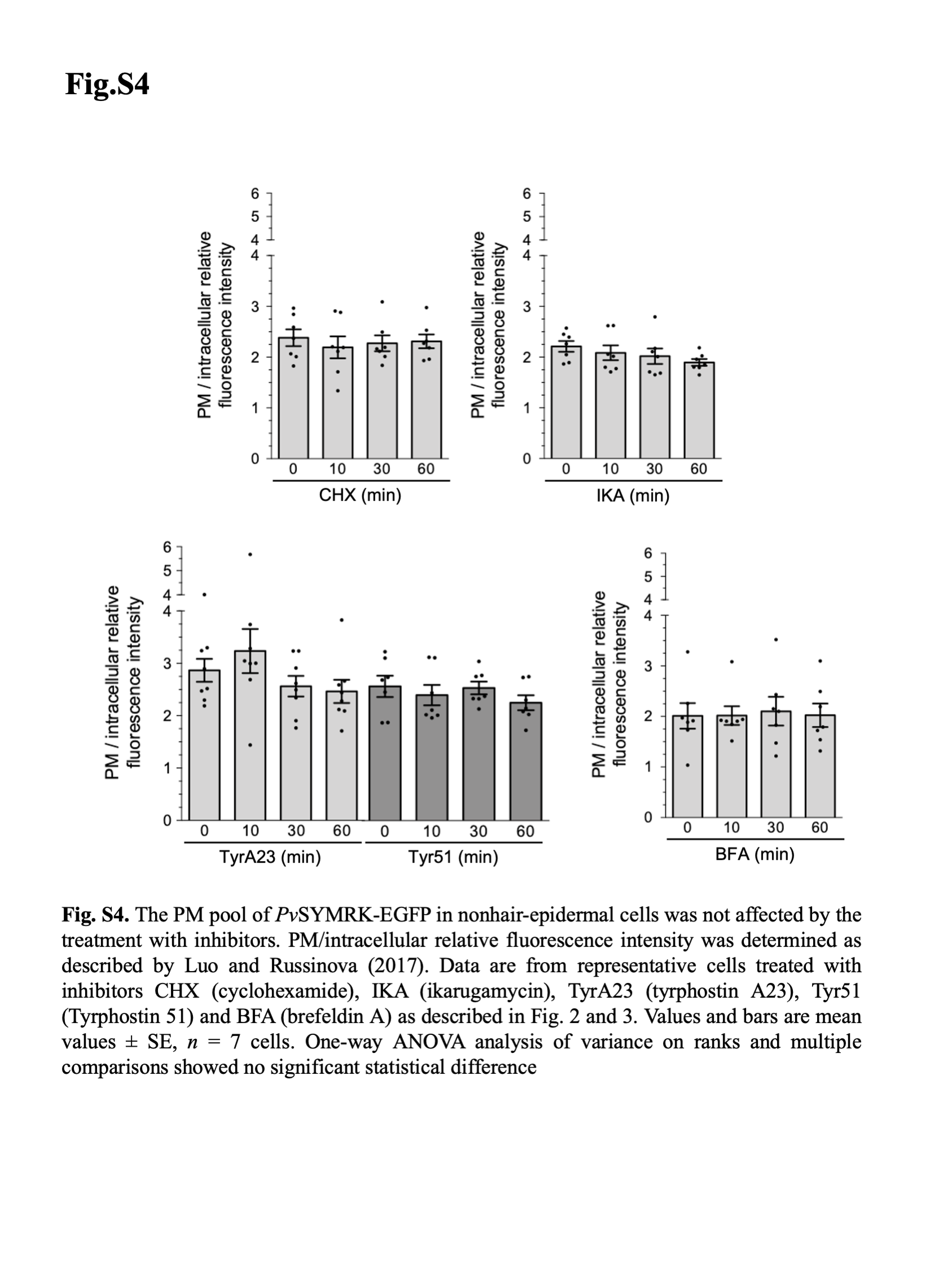

Supplement: Supplementary file 5 — Supplementary file5 Fig. S4 The PM pool of PvSYMRK-EGFP in nonhair-epidermal cells was not affected by the treatment with inhibitors. PM/intracellular relative fluorescence intensity was determined as described by Luo and Russinova (2017). Data are from representative cells treated with inhibitors cyclohexamide (CHX), ikarugamycin (IKA), tyrphostin A23 (TyrA23), tyrphostin 51 (Tyr51) and brefeldin A (BFA) as described in Fig. 2 and 3. Values and bars are mean values ± SE, n = 7 cells. One-way ANOVA analysis of variance on ranks and multiple comparisons (Tukey’s method) showed no significant statistical difference (TIFF 11119 KB) [file 425_2023_4116_MOESM5_ESM.tiff]
